# Cancer-associated fibroblasts and immune remodeling in laryngeal squamous cell carcinoma: evidence and translational perspectives

**DOI:** 10.3389/fimmu.2026.1848015

**Published:** 2026-08-20

**Authors:** Wei-Zhe Hong, Guan-Jiang Huang, Qi-Ping Luo, Biao-Qing Lu

**Affiliations:** 1 Department of Otorhinolaryngology Head and Neck Surgery, Zhongshan Hospital of Traditional Chinese Medicine, Affiliated to Guangzhou University of Traditional Chinese Medicine, Zhongshan, Guangdong, China; 2 The Tenth Clinical Medical College of Guangzhou University of Traditional Chinese Medicine, Zhongshan, Guangdong, China

**Keywords:** cancer-associated fibroblasts, immune evasion, immunotherapy resistance, laryngeal squamous cell carcinoma, organ preservation, single-cell RNA sequencing, spatial transcriptomics, tumor microenvironment

## Abstract

Laryngeal squamous cell carcinoma (LSCC) remains a clinically challenging malignancy of the head and neck. Because organ preservation is a major therapeutic objective in LSCC, stromal biology is of particular clinical relevance. Stromal factors influence local disease control, functional outcomes, and treatment resistance. Cancer-associated fibroblasts (CAFs) are dynamic regulators of extracellular matrix remodeling, immune exclusion, angiogenesis, and therapeutic resistance. However, LSCC-specific evidence remains fragmented, and many mechanistic concepts are still extrapolated from head and neck squamous cell carcinoma (HNSCC) and hypopharyngeal squamous cell carcinoma (HPSCC). This review adopts an evidence-stratified framework that distinguishes direct LSCC evidence from translatable cross-site evidence and hypothesis-generating inferences. We propose a provisional five-state model for LSCC that includes myofibroblastic, inflammatory, antigen-presenting, extracellular matrix-remodeling, and putative immune-trapping CAF programs. Direct LSCC studies support the presence of early stromal activation along the leukoplakia-to-carcinoma continuum and the enrichment of *FAP*-positive and *POSTN*-positive fibroblastic programs in metastatic disease. Associations involving stromal PD-L1 expression and altered CAF-derived exosomal microRNA cargo have also been reported. Translatable evidence from HNSCC implicates TGF-β- and CXCL12-dependent T-cell exclusion together with Gal9-mediated T-cell dysfunction. Additional mechanisms include IL-6/JAK/STAT3-driven myeloid skewing and NOX4-dependent stabilization of the myofibroblastic phenotype. We integrate these findings into a spatially informed conceptual framework encompassing the invasive front, the perivascular compartment, the cartilage interface, and the lymphovascular niche. We also discuss biomarker-guided therapeutic opportunities, including CAF normalization, *FAP*-directed approaches, stromal signaling blockade, extracellular matrix-targeted strategies, and interference with extracellular vesicle-mediated signaling. This review thereby establishes an evidence-stratified framework for CAF heterogeneity in LSCC and clarifies the translational value and current limitations of stroma-targeted strategies. It further provides a structured foundation for hypothesis-driven clinical and experimental investigation in laryngeal cancer.

## 1 Introduction

LSCC remains one of the most clinically consequential malignancies of the head and neck. Recent global estimates confirm a substantial worldwide burden of laryngeal cancer (1, 2). Incidence and mortality remain high across regions. Despite advances in surgery, radiotherapy, and systemic treatment, long-term survival for advanced LSCC remains suboptimal.

A key feature of LSCC is the clinical importance of organ preservation. Landmark trials demonstrated that selected patients with advanced laryngeal cancer are amenable to larynx-preserving chemoradiotherapy (3, 4), with overall survival non-inferior to that achieved with total laryngectomy. In this setting, the biological determinants of local control extend beyond recurrence risk alone. Failure of organ preservation may ultimately necessitate total laryngectomy, resulting in permanent loss of natural voice and impairment of both swallowing and airway protection.

The LSCC tumor microenvironment has increasingly been recognized as a determinant of progression and therapeutic response. Direct LSCC single-cell, clinicopathologic, and extracellular vesicle studies now support clinically relevant stromal heterogeneity. These studies additionally document metastatic fibroblast remodeling, stemness-associated tumor states, stromal PD-L1 associations, and abnormal CAF-derived RNA cargo (5–10). Parallel advances in squamous carcinoma atlases and related head and neck studies have further refined state-based views of fibroblasts and spatial immune remodeling (11–18). Among stromal populations, CAFs are increasingly implicated in extracellular matrix remodeling, neutrophil and myeloid skewing, immune regulation, and resistance to radiotherapy and immune checkpoint blockade (14, 19, 20). More broadly, foundational work in fibroblast biology together with single-cell and spatial technologies has transformed CAFs biology from a marker-based concept into a dynamic, state-based framework (21–29).

Yet LSCC-specific synthesis remains incomplete. Existing CAF reviews mainly address pan-HNSCC or broader solid tumor biology. They do not fully integrate single-cell atlases, spatial architecture, immune remodeling circuits, and translational vulnerabilities into an LSCC-centered model. The available LSCC literature is heterogeneous, comprising observations derived directly from LSCC cohorts as well as findings inferred from HNSCC, HPSCC, or pan-cancer studies. This distinction carries important interpretive implications. Overextension of translatable evidence may overstate the maturity of LSCC stromal biology. Excessive conservatism may obscure actionable mechanistic themes supported by convergent cross-disease data.

Accordingly, this review has four aims. First, we define an evidence-stratified framework for CAF states in LSCC. Second, we examine how CAF programs remodel antitumor immunity. Third, we connect CAF biology to invasion, nodal dissemination, organ preservation failure, and immunotherapy resistance. Fourth, we identify biomarker anchored therapeutic opportunities and research priorities while distinguishing direct and translatable evidence.

## 2 Literature identification strategy and evidence framework

### 2.1 Narrative review approach and literature identification

This review was conducted as an evidence-stratified narrative review rather than a formal systematic review. Its aim was to provide a mechanistically oriented and translationally relevant synthesis of the emerging CAF literature in LSCC. Literature was identified through targeted searches of PubMed, Web of Science, and Embase. Search terms covered disease context, stromal biology, immune regulation, and advanced profiling technologies. Representative terms included laryngeal squamous cell carcinoma, cancer-associated fibroblast, single-cell RNA sequencing, spatial transcriptomics, PD-L1, TGF-β, *CXCL12*, NOX4, and extracellular vesicle. The review strategy was designed to prioritize studies with the highest direct relevance to LSCC biology and stromal-immune interactions. Study selection prioritized original investigations directly examining LSCC, particularly those conducted in human tissue specimens or clinically annotated patient cohorts. Additional weight was accorded to studies employing cell-resolved or spatially resolved analytical approaches. These approaches are especially informative for defining CAF heterogeneity, immune geography, and niche-level organization. Mechanistic studies in broader HNSCC and closely related HPSCC were incorporated when they offered biologically plausible insight applicable to LSCC. Seminal pan-cancer studies were included selectively. They were used mainly to support conserved stromal or immune mechanisms when LSCC specific evidence was limited. Throughout the review, this hierarchy was used to separate direct observations from contextual support. It was also used to avoid overextending disease-specific conclusions.

Because the field remains relatively young, older foundational CAF and tumor microenvironment studies were retained when they remained mechanistically informative. Throughout the manuscript, cautious language is used for findings derived mainly from computational inference, bulk tissue association, or cross-site extrapolation.

### 2.2 Evidence grading used in this review

To enhance interpretability, the evidence in this review was organized into a four level grading framework. This framework separates disease-specific observations from broader mechanistic inference. Level A comprises direct LSCC evidence. This includes human LSCC studies with single-cell, spatial, multiplex protein, functional, or clinicopathologic data relevant to CAF biology and immune regulation. These studies form the strongest foundation for conclusions specific to LSCC and are therefore given the greatest interpretive weight throughout the review. Level B includes LSCC adjacent evidence. This includes premalignant laryngeal lesions, metastatic LSCC, supraglottic specific LSCC subsets, and non cell resolved stromal analyses in LSCC. These studies are informative but do not fully define CAF state, spatial organization, or functional impact. Although these data remain relevant to the broader understanding of stromal remodeling in laryngeal malignancy, their inferential scope is more limited than that of directly resolved LSCC datasets. Level C refers to HNSCC- or HPSCC-translatable evidence, comprising mechanistic, single-cell, or spatial studies in anatomically and biologically related squamous carcinomas. These studies offer plausible insight into CAF programs, stromal-immune interactions, or therapeutic vulnerabilities relevant to LSCC, but have not yet been directly validated in LSCC tissues. Finally, Level D comprises pan-cancer mechanistic support, namely foundational studies from other tumor types that are cited to illustrate conserved stromal or immune principles without implying direct proof in LSCC. This graded approach was not adopted as a formal scoring system for study quality. Rather, it served as a conceptual tool to maintain transparency regarding the degree of biological proximity between the cited evidence and the specific questions addressed in LSCC.

Throughout the review, stronger verbs are reserved for Level A evidence or exceptionally convergent multiplatform data. More cautious verbs are used for indirect evidence. Representative examples are summarized in **Table 1**.

**Table 1:** Evidence framework and representative studies relevant to CAF biology in LSCC.

Study	Disease context	Platform/study type	CAF-relevant insight	Evidence level	Main limitation for LSCC interpretation
Song et al. (5)	LSCC and laryngeal lesion ecosystem	Single-cell atlas/TME mapping	Supports stromal heterogeneity and laryngeal ecosystem remodeling	A/B	Fibroblast states not fully resolved into a modern CAF taxonomy
Sun et al. (6)	Metastatic LSCC	scRNA-seq	Node-positive tumors enriched for extracellular matrix and migration-associated fibroblast programs; *FAP*^+^/*POSTN*^+^ programs associated with nodal disease	A	Metastatic context may not represent all LSCCs
Li et al. (7)	LSCC	scRNA-seq	Identifies cancer stem cell-like tumor programs and stromal interaction context relevant to stemness	A	Not primarily designed as a CAF study
Vassilakopoulou et al. (8)	LSCC	Clinicopathologic/IHC	Stromal PD-L1 associates with immune infiltrates	A	Stromal PD-L1 is not cell-type specific for CAFs
Verro et al. (9)	Advanced LSCC	Clinicopathologic/PD-L1 assessment	Supports clinical relevance of stromal and tumor PD-L1 context	A/B	Preliminary clinical series; limited cell resolution
Wu et al. (10)	Supraglottic LSCC	CAF-derived exosomal microRNA profiling	Direct LSCC evidence for altered CAF-derived extracellular vesicle cargo	A/B	Small cohort; mainly descriptive and bioinformatic
Puram et al. (11)	HNSCC	scRNA-seq	Defines ecosystem heterogeneity, partial epithelial-to-mesenchymal transition programs, and fibroblast relevance	C	Not LSCC-specific
Ji et al. (12)	Human squamous carcinoma	Spatial/multimodal analysis	Establishes spatial architecture of fibroblast-rich invasive niches	C/D	Broader squamous carcinoma framework, not LSCC-specific
Li et al. (13)	HNSCC	Spatial + single-cell transcriptomics	Identifies an MHC-I-high/Gal9^+^ immune-trapping CAF program	C	Requires direct LSCC validation
Cai et al. (18)	HPSCC	scRNA-seq	Shows pro-invasive *FAP*^+^ CAF states and endothelial/macrophage crosstalk	C	Adjacent site; anatomic translation incomplete
Fayette et al. (62)	Recurrent/metastatic HNSCC	Randomized phase II conference abstract	Early clinical support for CAF normalization with setanaxib plus pembrolizumab	C	HNSCC-wide cohort; abstract-level evidence
Fang et al. (37)	Vocal fold leukoplakia	Functional/IHC/*in vitro* and *in vivo*	FAP^+^/αSMA^+^ CAF-like fibroblasts suppress CD8^+^ T cells and recruit Tregs through IL-6/JAK2/STAT3	B	Premalignant and LSCC-adjacent, not invasive LSCC
Zhang et al. (35)	OSCC primary tumors and lymph nodes	scRNA-seq/functional validation	COMP^+^ mCAF2 in metastatic lymph nodes shows enhanced ECM activity linked to ENE	C	Oral cavity, not larynx
Zhao et al. (36)	LSCC	Mechanistic molecular study	HOXA10-AS/miR-29b-3p/ITGA6 axis links matrix-associated integrin signaling to malignant progression and oxidative resistance	A/B	Tumor-cell-centered, not CAF-resolved
Chen et al. (56)	OSCC	scRNA-seq/spatial/functional assays	FAP^+^ fibroblasts promote C1QC^+^ macrophage infiltration via WNT2/β-catenin and exacerbate T-cell exhaustion	C	Strongly translatable but not LSCC-specific
Feng et al. (61)	OSCC	Review/mechanistic synthesis	EV secretion is proposed as a modifiable TME process; CBD may affect Wnt/β-catenin and STAT3-related EV biology	C/D	Review-level and indirect for LSCC

## 3 Conceptual framework for CAFs in LSCC

### 3.1 Origins of CAFs in LSCC

In LSCC, CAFs most likely arise from resident laryngeal fibroblasts, pericytes, and bone marrow-derived mesenchymal progenitors (21, 26, 30). These cells are activated by tumor-derived signals. Endothelial-to-mesenchymal transition and adipocyte transdifferentiation have been described in other tumor types. Their contribution to LSCC remains poorly characterized. TGF-β, platelet-derived growth factor, inflammatory cytokines, and reactive oxygen species are recognized inducers of fibroblast activation and myofibroblastic differentiation.

A potentially site-specific feature of LSCC is the presence of hyaline cartilage and fibrous perichondrium within the larynx. Fibroblasts at the cartilage-perichondrium interface may experience distinct biomechanical cues. They may also face injury patterns not seen in the oral cavity or oropharynx. These anatomic constraints may help shape extracellular matrix-dense fibroblastic programs, particularly in tumors approaching or invading laryngeal cartilage. Although this concept remains inferential, it offers a plausible biological explanation for why matrix-remodeling CAF programs may be especially relevant in LSCC.

### 3.2 Marker systems and their limitations

No single protein uniquely identifies CAFs in LSCC or any other tumor type (21, 26). Commonly used markers include fibroblast activation protein alpha (*FAP*), α-smooth muscle actin (αSMA), platelet-derived growth factor receptor beta (PDGFRβ), collagen type I alpha 1 (*COL1A1*), periostin (*POSTN*), fibroblast-specific protein 1 (FSP1), and vimentin. However, each marker has limitations. αSMA preferentially labels myofibroblastic CAFs and is also expressed by vascular smooth muscle cells and pericytes. *FAP* is more selective for tumor-activated fibroblasts, yet it is not uniformly expressed across all CAF states. PDGFRβ may enrich pericyte-like or mesenchymal stromal populations but is not restricted to CAFs. CAF characterization in LSCC should therefore rely on marker panels and spatial context. When possible, such characterization should be integrated with transcriptomic or multiplex imaging data, rather than relying on single-marker immunohistochemistry alone.

### 3.3 From marker-based fibroblasts to state-based CAF programs

A major conceptual advance in stromal biology has been the shift from viewing CAFs as a single cell type to recognizing them as dynamic, context-dependent, and partly interconvertible programs (22, 27–29). Across tumor types, single-cell studies consistently identify at least myofibroblastic and inflammatory fibroblastic states, with additional antigen-presenting, matrix-remodeling, stress-responsive, or immune-regulatory states depending on tissue context (23–25, 31–33). Spatial studies further indicate that these programs are not randomly distributed. Myofibroblastic states often localize to invasive or perivascular regions, whereas inflammatory states may be enriched in immune-interacting stromal zones.

In LSCC, the current evidence is still insufficient to support a definitive disease-specific CAF taxonomy. Nevertheless, available data are sufficient to support a provisional, evidence-stratified framework of CAF programs relevant to LSCC biology and therapy response.

### 3.4 A provisional evidence-stratified CAF framework for LSCC

Drawing on direct LSCC studies and translatable HNSCC/HPSCC data, we propose five recurrent CAF programs relevant to LSCC biology (**Table 2**). These should be viewed as functional states rather than rigid lineages.

**Table 2:** Provisional evidence-stratified CAF framework in LSCC.

CAF program	Hallmark markers/pathways	Dominant biological functions	Putative spatial niche	Evidence strength in LSCC	Key translational implication
myCAF	*ACTA2*, *TAGLN*, *POSTN*, *MMP11*, *COL1A1*; TGF-β signaling	Extracellular matrix deposition, tissue stiffening, CD8^+^ T-cell exclusion, therapy resistance	Invasive front, fibrotic stroma, perivascular zones	Moderate direct ^+^ strong translatable	Strong rationale for NOX4 and TGF-β-directed normalization
iCAF	*IL6, IL8, CXCL1, CXCL5, CXCL12;* inflammatory secretome	Myeloid skewing, cytokine amplification, T-cell trapping, inflammatory remodeling	Immune-interacting stromal niches, perivascular regions	Limited-to-moderate direct + strong translatable	Supports *IL-6/JAK/STAT3 and CXCL12/CXCR4* targeting
apCAF	*MHC-II*, *CD74*, antigen-processing genes	Context-dependent antigen presentation, potential regulatory T-cell induction	Immune-enriched stromal compartments	Indirect/weakly validated	Requires direct LSCC validation before therapeutic use
ecmCAF	*FAP, COL11A1, POSTN, COMP, FN1, MMPs, LOX/LOXL2, fibrillar collagen programs*	Matrix deposition, degradation, fiber alignment, collagen crosslinking, integrin/mechanosignaling, invasion, immune exclusion, lymphovascular niche conditioning	Invasive front, cartilage interface, perivascular and lymphovascular regions, metastatic nodal stroma	Moderate direct/adjacent + strong translatable	Candidate state for FAP-directed, ECM-targeted, and macrophage-interaction-targeted strategies
Putative immune-trapping CAF	*MHC-I-high, CXCL9/10/12, LGALS9*	Functional CD8^+^ T-cell suppression, immune trapping, checkpoint-like stromal signaling	Immune-excluded stromal regions	Hypothesis-generating in LSCC; strong HNSCC support	High-priority validation target for stromal checkpoint combinations

#### 3.4.1 Myofibroblastic CAFs

Myofibroblastic CAFs (myCAFs) are characterized by high expression of *ACTA2*, *TAGLN*, *POSTN*, *MMP11*, and fibrillar collagens, with strong enrichment for TGF-β signaling and contractile cytoskeletal programs (11, 15, 24, 25, 34). Among the proposed states, myCAFs are the most consistently supported in squamous carcinomas and likely represent the dominant fibrotic barrier-forming program in LSCC. Their predicted roles include extracellular matrix deposition, matrix stiffening, immune exclusion, and reduced intratumoral drug penetration.

#### 3.4.2 Inflammatory CAFs

Inflammatory CAFs (iCAFs) express *IL-6, IL-8*, *CXCL1, CXCL5, CXCL12*, and related inflammatory mediators, often coupled to protease activity and glycolytic or stress-response programs (20, 31, 32). In metastatic LSCC, *CXCL12*-enriched fibroblastic populations and fibroblast programs associated with nodal disease support the biological relevance of an iCAF-like state, although its exact transcriptomic boundaries remain incompletely defined in LSCC (6). iCAFs are especially relevant to myeloid skewing, cytokine amplification, and treatment-associated inflammatory remodeling.

#### 3.4.3 Antigen-presenting CAFs

Antigen-presenting CAFs (apCAFs) are defined by MHC class II, *CD74*, and antigen-processing genes (33). Their function in LSCC remains unresolved. In other tumors, they have been linked to nonproductive antigen presentation and regulatory T-cell induction, although context-dependent immune-stimulatory effects have also been proposed. At present, apCAFs remain a plausible but weakly validated candidate state in LSCC that awaits direct confirmation.

#### 3.4.4 Extracellular matrix-remodeling CAFs

Extracellular matrix-remodeling CAFs (ecmCAFs) are enriched for FAP, COL11A1, POSTN, COMP, FN1, MMPs, fibrillar collagens, and LOX/LOXL2-related matrix-crosslinking programs. Functionally, they remodel the tumor scaffold through collagen and fibronectin deposition, matrix degradation and realignment, periostin- and integrin-dependent mechanosignaling, and collagen crosslinking. These processes can stiffen the invasive front and promote epithelial migration and lymphovascular spread. They may also compress vessels, impair drug distribution, and generate matrix-dense stromal tracks that limit CD8^+^ T-cell entry. This program likely overlaps partially with myCAFs but remains useful for emphasizing invasive-front biology, cartilage-interface remodeling, and lymphovascular niche conditioning. Direct LSCC support comes from the metastatic atlas showing enrichment of FAP^+^ and POSTN^+^ fibroblast programs in node-positive disease (6). Adjacent OSCC evidence further shows that metastatic-lymph-node COMP^+^ myofibroblastic CAFs display enhanced extracellular matrix activity linked to extranodal extension (35). In LSCC, the HOXA10-AS/miR-29b-3p/ITGA6 axis also links integrin-associated tumor-matrix signaling to oxidative resistance and malignant progression, although this evidence is tumor-cell-centered rather than CAF-specific (36).

#### 3.4.5 Putative immune-trapping CAFs

A recently described HNSCC fibroblast state characterized by high MHC class I, *CXCL9, CXCL10, CXCL12*, and *Gal9* appears enriched in immune-excluded regions and inversely correlates with TCF1^+^*GZMK*^+^ CD8^+^ T cells (13). This program has not yet been directly demonstrated in LSCC and therefore remains a hypothesis-generating candidate relevant to LSCC, not yet an established subtype. If validated in laryngeal tissue, it would constitute a distinct stromal checkpoint-like program with major therapeutic implications.

## 4 CAF states, spatial niches, and cell-cell communication in LSCC

### 4.1 CAF emergence along the leukoplakia-to-carcinoma trajectory

An important LSCC-adjacent observation from single-cell studies is that fibroblast activation may begin before overt invasive carcinoma. Analyses spanning benign vocal cord lesions, vocal cord leukoplakia, and LSCC suggest that activated stromal programs emerge during high-grade dysplasia rather than appearing only after established invasion (5, 17). In these datasets, ligand-receptor inference nominated candidate early epithelial-stromal communication axes. These included *JAG1*-*NOTCH4* and *CXCL5*-related signaling between high-risk epithelial programs and activated fibroblasts. While these findings remain primarily computational and require orthogonal validation, they support the concept that CAF-associated immune exclusion and matrix remodeling may begin during premalignant progression.

A key LSCC-adjacent functional study further strengthens this early-stromal model. Fang et al. identified FAP^+^ and αSMA^+^ CAF-like fibroblasts in human vocal fold leukoplakia. These fibroblasts secreted IL-6 and TGF-β, maintained an IL-6/JAK2/STAT3 autocrine loop, induced CD8^+^ T-cell apoptosis and exhaustion, and recruited regulatory T cells. IL-6R blockade with tocilizumab suppressed fibroblast activation *in vitro* and delayed leukoplakia progression *in vivo* (37). These findings support the view that fibroblast-mediated immune remodeling may precede invasive LSCC, although longitudinal validation in laryngeal cohorts remains needed.

This possibility is clinically relevant in LSCC because the vocal cord leukoplakia-to-carcinoma continuum is amenable to surveillance and local intervention. If stromal activation proves to be an early event, fibroblast-centered biomarkers may help identify lesions at highest risk of malignant transformation.

### 4.2 CAF programs in metastatic and treatment-shaped LSCC ecosystems

**Figure 1:**
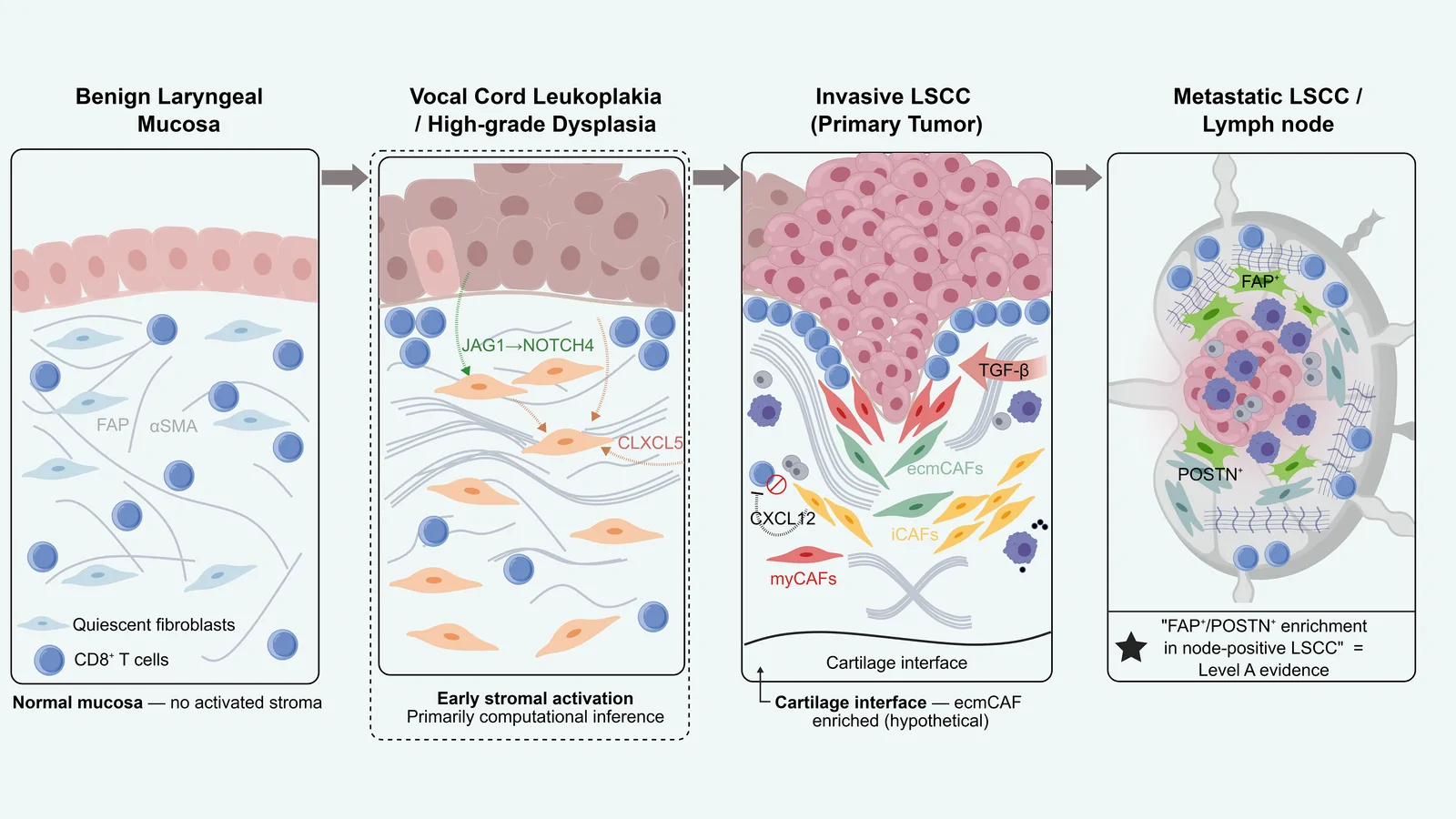
Emergence of fibroblast activation along the leukoplakia-to-LSCC continuum. Proposed model of stromal evolution from benign laryngeal mucosa and vocal cord leukoplakia/high-grade dysplasia to invasive and metastatic LSCC. Early stromal activation, epithelial-stromal communication, increasing myCAF/ecmCAF complexity, and progressive immune remodeling are illustrated. Direct LSCC evidence and translatable mechanisms are indicated separately. Created with BioGDP.com (38).

Direct LSCC evidence is stronger in metastatic disease. Single-cell profiling of metastatic and non-metastatic LSCC showed enrichment of fibroblast populations in node-positive tumors (6). These populations displayed extracellular matrix-regulatory, migration-promoting, and stromal activation signatures. Higher proportions of *FAP*-positive and *POSTN*-positive fibroblastic programs in primary tumors were associated with lymphatic metastasis. This supports a link between matrix-active fibroblasts and nodal dissemination. These observations collectively point toward a stepwise model of stromal activation and immune remodeling from premalignant laryngeal lesions to invasive and metastatic LSCC (**Figure 1**).

By contrast, direct post-immunotherapy LSCC single-cell data remain limited. Much of the evidence for treatment-shaped CAF ecosystems instead comes from broader HNSCC or pan-cancer studies. These studies show that residual disease after therapy may become enriched in fibrotic, myCAF-like programs and stromal barriers associated with poor T-cell penetration (39). These observations are highly relevant to LSCC, although direct proof in laryngeal tissue is still lacking.

An additional unresolved issue is anatomic subsite heterogeneity. Supraglottic tumors arise in a lymphatic-rich environment and may be predisposed to inflammatory and lymphovascular stromal remodeling. Glottic tumors, by contrast, may be shaped more strongly by mechanical stress, phonatory tissue architecture, and lower baseline lymphatic density. Comparative single-cell studies across glottic, supraglottic, and transglottic LSCC are still lacking, and this gap likely contributes to current imprecision in LSCC stromal taxonomy.

### 4.3 Spatial niches in LSCC

Although direct LSCC spatial transcriptomic studies remain sparse, current evidence supports four functionally relevant CAF-associated niches.

#### 4.3.1 The invasive-front fibrotic niche

This niche is characterized by dense myCAF/ecmCAF accumulation, collagen deposition, lysyl oxidase-dependent matrix crosslinking, and relative exclusion of cytotoxic lymphocytes (12, 40, 41). In LSCC, this niche is likely important for local invasion and for the partial epithelial-to-mesenchymal transition programs associated with aggressive tumor behavior.

#### 4.3.2 The perivascular immunoregulatory niche

Perivascular fibroblasts and iCAF-like programs may regulate immune trafficking through *CXCL12* gradients, endothelial adhesion molecule modulation, and paracrine communication with endothelial cells (18, 42, 43). This niche is conceptually important for understanding why T cells may be present in stromal compartments yet fail to enter tumor nests.

#### 4.3.3 The cartilage-interface fibrotic niche

A site-specific LSCC niche likely exists at the tumor-cartilage boundary or perichondrial interface. We hypothesize that this region favors ecmCAF programs with strong matrix deposition, tissue stiffening, and invasion-promoting behavior. This concept is supported indirectly by laryngeal anatomy and by the clinical importance of cartilage invasion in LSCC staging, but it remains to be directly mapped by high-resolution spatial technologies.

#### 4.3.4 The lymphovascular or pre-metastatic niche

CAF-mediated remodeling of lymphatic channels and perilymphatic stroma, potentially through VEGF-C, VEGF-D, prostanoids, and matrix reorganization, may facilitate nodal dissemination (18, 44). Direct LSCC spatial evidence remains limited, but the association between *FAP*^+^ and *POSTN*^+^ fibroblast programs and nodal disease supports this possibility (6).

### 4.4 A CAF-centered cell-cell communication map

Across LSCC and translatable HNSCC datasets, several CAF-centered communication axes recur.

First, the epithelial-CAF axis is driven by TGF-β, epidermal growth factor-family ligands, and matrix-associated signals that promote reciprocal tumor-stromal activation and epithelial plasticity (45–47). Second, the CAF-CD8^+^ T-cell axis involves matrix barrier formation, *CXCL12*-mediated marginal trapping, and likely checkpoint-like stromal signaling that limits cytotoxic infiltration and persistence (13, 42, 43, 47). Third, the CAF-myeloid and antigen-presenting cell axis includes *IL-6/JAK/STAT3*-dependent myeloid skewing together with tolerogenic dendritic-cell-oriented signals that amplify local immunosuppression (19, 48–50). Fourth, CAFs likely participate in metabolic crosstalk, including lactate-rich stromal circuits that influence immune-cell fitness and local acidity (51). Finally, these observations fit within a broader conceptual framework in which TGF-β-regulated and lectin-glycan-regulated immune circuits cooperate in shaping suppressive tumor niches (52, 53).

Importantly, many of these interactions are currently derived from ligand-receptor inference rather than direct perturbation experiments in LSCC. Computationally inferred communication should therefore be interpreted as a prioritization framework rather than final proof of mechanism.

### 4.5 Methodological caveats specific to LSCC

LSCC presents specific technical challenges for stromal profiling. First, the cartilaginous and fibrous architecture of the larynx complicates tissue dissociation. This may systematically underrepresent stromal populations in single-cell RNA sequencing. Second, prior radiotherapy or chemoradiotherapy can induce dense fibrosis, necrosis, and RNA degradation, all of which affect tissue quality and cell-state recovery. Third, treatment timing matters, as therapy-naïve primary tumors, post-radiation residual tumors, salvage laryngectomy specimens, and recurrent or metastatic lesions likely harbor fundamentally different CAF compositions. Fourth, marker ambiguity remains a practical issue in routine pathology, as αSMA-rich stroma is not equivalent to total CAF burden. Finally, spatial deconvolution algorithms can be confounded in highly fibrotic tissues unless supported by multiplex protein-level validation.

Future LSCC studies should prioritize paired treatment-naïve and post-treatment samples. They should also use cartilage-adapted dissociation protocols and explicit subsite annotation. Orthogonal validation with multiplex imaging or high-definition spatial transcriptomics is also needed (12, 24, 54).

## 5 Mechanisms of CAF-mediated immune remodeling in LSCC

**Figure 2:**
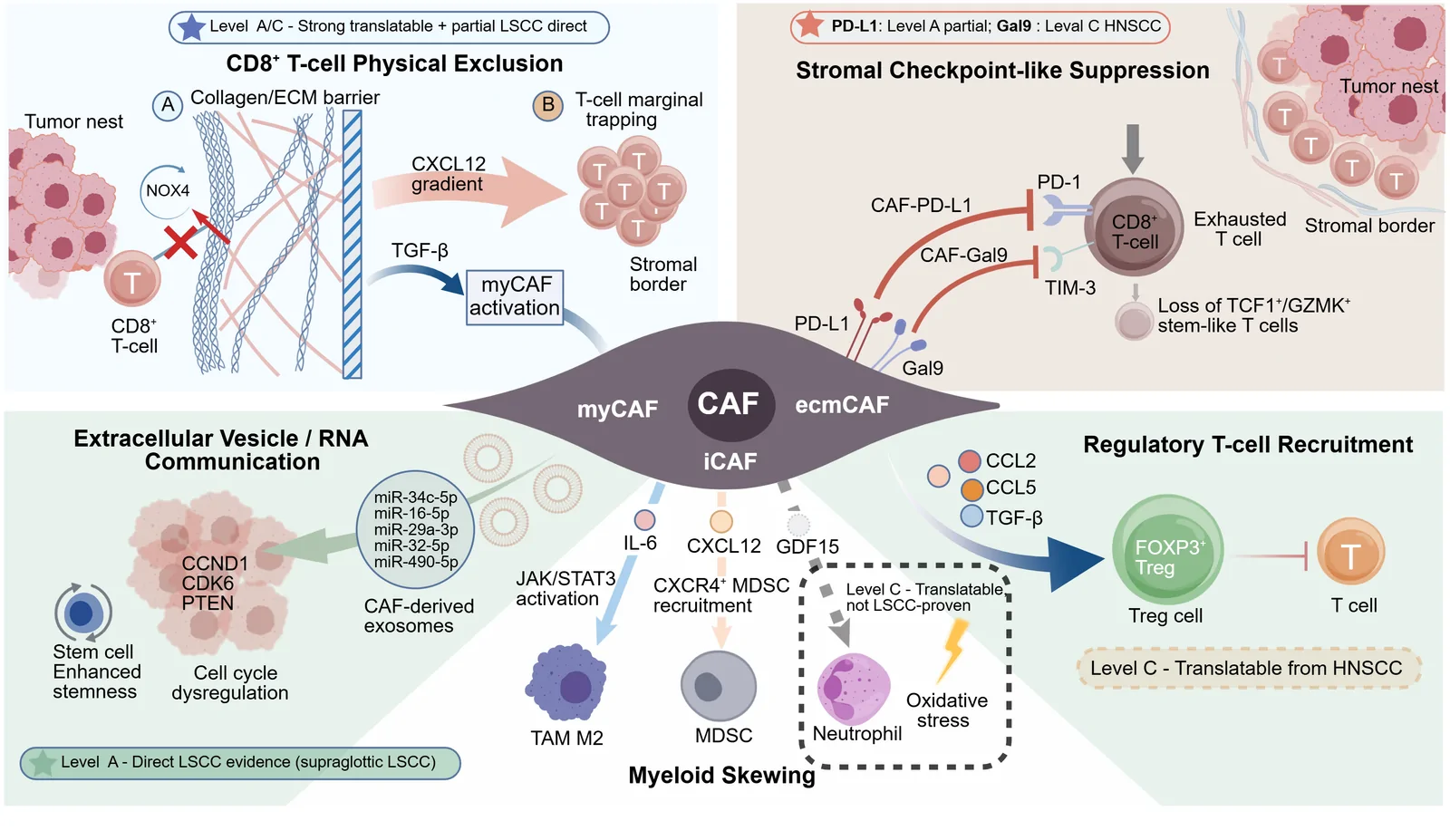
Mechanisms of CAF-mediated immune remodeling in LSCC. Schematic summary of the major mechanisms by which cancer-associated fibroblasts (CAFs) remodel antitumor immunity in laryngeal squamous cell carcinoma (LSCC), including CD8^+^ T-cell exclusion, stromal checkpoint-like suppression, recruitment of suppressive immune cells, impairment of dendritic-cell and natural killer-cell responses, and extracellular vesicle-mediated RNA signaling. Direct LSCC evidence and translatable mechanisms are indicated separately. Created with BioGDP.com (38).

The major CAF-mediated mechanisms of immune remodeling in LSCC are summarized in **Figure 2**.

### 5.1 Physical exclusion and functional impairment of CD8^+^ T cells

CAF-mediated restriction of CD8^+^ T-cell immunity occurs through at least two linked processes, namely physical exclusion and functional impairment.

Physical exclusion is strongly associated with myCAF-driven extracellular matrix deposition. Activated fibroblasts deposit collagens, fibronectin, and related matrix proteins while concurrently promoting matrix crosslinking (40, 41). These changes increase tissue stiffness and reduce lymphocyte motility. TGF-β is a central upstream regulator of this process, reinforcing myofibroblastic differentiation and collagen synthesis (46, 47, 52). In non-LSCC models, stromal TGF-β can convert inflamed tumors into immune-excluded lesions and attenuate response to anti-PD-L1 therapy. NOX4-dependent stabilization of the myofibroblastic phenotype appears to reinforce this fibrotic barrier (34, 55). These findings are highly translatable to LSCC, particularly because radiotherapy itself can intensify tissue fibrosis.

A complementary mechanism is *CXCL12*-mediated T-cell trapping (42, 43). Fibroblast-derived *CXCL12* retains T cells in stromal or peritumoral compartments. This limits entry into malignant epithelial nests. This mechanism has not yet been functionally demonstrated in LSCC with the same rigor as in other tumor types. Nevertheless, CXCL12-rich fibroblast programs in metastatic LSCC and inflammatory CAF states in HNSCC make this axis especially plausible.

Functional impairment is exemplified by the HNSCC-derived immune-trapping CAF program enriched for MHC-I and Gal9 (13). In that setting, Gal9 expressed on stromal fibroblasts engages TIM3 on CD8^+^ T cells and is associated with depletion of stem-like TCF1^+^*GZMK*^+^ cytotoxic precursors needed for durable immunotherapy response. Whether an identical program operates in LSCC remains unresolved. However, it remains a high-priority stromal checkpoint hypothesis for future validation.

### 5.2 Stromal checkpoint-like signaling: PD-L1, Gal9, and cell-of-origin uncertainty

The concept that stromal cells may participate directly in immune checkpoint regulation has important implications for LSCC. In LSCC cohorts, stromal PD-L1 expression has been reported and correlates with immune infiltrates and clinicopathologic variables, including nodal status and inflammatory measures in some series (8, 9). Stromal PD-L1 positivity on routine immunohistochemistry does not prove CAF-specific PD-L1 expression. Macrophages, dendritic cells, endothelial cells, and other stromal cells may also contribute.

A cautious interpretation is more appropriate. The stromal checkpoint landscape appears clinically relevant in LSCC. It likely includes fibroblastic contributions that require higher-resolution mapping. In HNSCC and related settings, selective interference with CAF-associated PD-L1 signaling has been linked to restoration of T-cell effector function and reduction of immunosuppressive cytokine profiles (14, 20). These data justify direct LSCC multiplex validation rather than immediate mechanistic certainty.

Gal9 represents a second checkpoint-like stromal ligand of interest. HNSCC single-cell and spatial studies indicate that Gal9-rich fibroblast programs are enriched in immune-excluded regions and inversely associated with stem-like CD8^+^ T-cell populations (13). Together, stromal PD-L1 and Gal9 support the broader concept of a stromal checkpoint program, but its exact cellular composition and relative contribution in LSCC remain to be determined.

### 5.3 Recruitment and education of suppressive immune cells

CAFs influence not only cytotoxic T cells but also the composition of suppressive immune populations. In HNSCC and other solid tumors, CAF-derived *CCL2*, *CCL5*, *TGF-β*, and related mediators support recruitment or local induction of *FOXP3*^+^ regulatory T cells (14, 34, 44). This process may be particularly relevant in LSCC lesions characterized by stromal immune enrichment but poor cytotoxic effector function.

CAFs also shape the monocyte-macrophage axis. IL-6 secreted by inflammatory fibroblasts activates *JAK*-*STAT3* signaling in infiltrating monocytes (49, 50). This promotes differentiation toward an immunosuppressive M2-like tumor-associated macrophage phenotype. *CXCL12* additionally promotes recruitment of *CXCR4*^+^ myeloid-derived suppressor cells, further suppressing T-cell activity through metabolic and redox mechanisms (42, 43).

Recent cross-site squamous carcinoma data provide a more specific CAF-macrophage circuit. In OSCC, single-cell, spatial, and functional analyses showed that FAP^+^ fibroblasts secrete WNT2 and activate β-catenin signaling in macrophages. This signaling promotes C1QC^+^ macrophage infiltration and M2-like immunosuppressive polarization, thereby aggravating CD8^+^ T-cell exhaustion (56). Related spatial studies in other tumors also indicate that FAP^+^ fibroblasts can cooperate with SPP1^+^ macrophages to promote desmoplastic extracellular matrix remodeling and immune exclusion (57). In LSCC, these findings should be interpreted as translatable rather than proven. Nevertheless, they suggest that FAP/POSTN-high ecmCAF regions should be evaluated together with C1QC^+^, SPP1^+^, CD163^+^, and CD206^+^ macrophage markers in future spatial studies.

A related emerging pathway is the CAF-*GDF15*-neutrophil axis. In HNSCC, CAF-derived *GDF15* promotes neutrophil infiltration and oxidative stress through *PI3K/AKT/STAT3*-related signaling (19). This mechanism is relevant to LSCC because oxidative stress, radiation response, and stromal remodeling are closely linked clinically. However, a direct CAF-resolved role for *GDF15* in human LSCC remains insufficiently established. It should currently be regarded as translatable rather than proven.

### 5.4 CAF-mediated impairment of dendritic-cell and NK-cell responses

Direct LSCC evidence regarding CAF effects on dendritic cells and natural killer cells is limited. Nonetheless, broader HNSCC and pan-cancer studies show that CAF-derived factors such as *WNT2*, prostaglandin E2, and IL-10 can suppress dendritic-cell maturation, reduce MHC-II expression, and bias antigen-presenting cells toward tolerogenic states (48, 49). Similar stromal programs may limit local priming of antitumor T-cell responses in LSCC.

Natural killer-cell suppression can also result from CAF-derived metalloproteinases that cleave NKG2D ligands from tumor cell surfaces, thereby reducing tumor susceptibility to NK-mediated cytotoxicity (44, 58). Whether these pathways are quantitatively important in LSCC, particularly after radiotherapy or within cartilage-adjacent fibrotic niches, remains unknown.

### 5.5 Metabolic and oxidative immunosuppression imposed by CAFs

CAFs contribute to immune dysfunction through altered metabolism and redox regulation. Under the reverse Warburg effect, fibroblasts adopt aerobic glycolysis and export lactate or pyruvate, thereby acidifying the extracellular milieu and impairing T-cell activation (51). CAF-driven matrix stiffening and vascular compression may further amplify hypoxia, HIF-1α signaling, and immune-metabolic suppression.

In HNSCC, *GDF15*-related stromal programs have been linked to oxidative stress and Nrf2-associated redox remodeling (19, 48). These observations suggest that CAF-rich LSCC may combine fibrotic exclusion with metabolic and oxidative suppression, a combination potentially relevant to both immunotherapy resistance and radioresistance. However, direct LSCC data on these redox circuits remain limited.

### 5.6 Extracellular vesicles and non-coding RNA circuits: established findings and open questions

Among the most disease-specific findings in LSCC is the identification of altered CAF-derived exosomal microRNA cargo. In a study of moderately differentiated supraglottic LSCC, next-generation sequencing of CAF-derived exosomes revealed a predominantly downregulated microRNA profile compared with exosomes from normal fibroblasts, including miR-34c-5p, miR-16-5p, miR-29a-3p, miR-32-5p, and miR-490-5p (10). Bioinformatic analysis linked these changes to cell-cycle regulators and tumor-promoting pathways involving targets such as *CCND1*, *CDK6*, and *PTEN*.

These findings provide direct LSCC evidence that CAF-derived extracellular vesicle cargo is abnormal and potentially relevant to tumor progression. At the same time, several important caveats merit consideration. First, the study was small and site-specific, focusing on supraglottic LSCC. Second, the identified microRNA network was largely inferential. Third, direct causal mapping of individual CAF-exosomal microRNAs to immune escape or cancer stemness in LSCC remains incomplete. The biological plausibility of such mechanisms is high, especially given the broader role of extracellular vesicles in tumor-stroma communication and the recognized contribution of stemness programs to therapy resistance (59, 60).

Recent OSCC-focused literature also highlights extracellular vesicle secretion as a modifiable tumor-microenvironment process and proposes that cannabidiol may influence EV biogenesis through Wnt/β-catenin, STAT3, and mitochondrial calcium-related pathways. This remains indirect for LSCC and is therefore cited only as contextual support for EV-targeted strategies (61).

The extracellular vesicle and RNA axis in LSCC therefore remains an early but promising mechanistic domain. Current evidence supports translational interest. However, it does not support deterministic claims about individual cargo species or immediate biomarker deployment.

## 6 CAFs in LSCC progression, metastasis, and therapy resistance

### 6.1 CAFs, epithelial plasticity, stemness, and local invasion

CAF-driven epithelial plasticity is a recurrent theme in squamous cancers. Through TGF-β, hepatocyte growth factor, and *CXCL12*/SDF-1, fibroblasts can induce epithelial-to-mesenchymal transition-related transcriptional programs, facilitate motility, and promote resistance to anoikis (26, 45). Single-cell analyses of HNSCC have identified partial epithelial-to-mesenchymal transition as a clinically adverse tumor state linked to invasive behavior and nodal dissemination (11, 45). LSCC likely shares this biology, particularly at the invasive front where tumor cells encounter extracellular matrix-dense, fibroblast-rich stroma.

Direct LSCC evidence for cancer stem cell-related programs also exists. Single-cell characterization of LSCC identified *SOX4*^+^/*CD44*^+^ stem-like populations and highlighted stromal interaction programs relevant to stemness maintenance (7). Direct spatial proof of a CAF-defined cancer stem cell niche in LSCC remains limited. However, current data support a role for fibroblast-rich microenvironments in tumor plasticity and invasion.

### 6.2 CAFs and lymphatic dissemination

Direct LSCC evidence for CAF-driven lymphangiogenesis remains limited, but several observations point in this direction. In metastatic LSCC, node-positive tumors contain increased *FAP*^+^ and *POSTN*^+^ fibroblast programs in primary lesions (6). In HNSCC and HPSCC, fibroblast-derived VEGF-C, VEGF-D, and prostanoid signaling support lymphatic remodeling and metastatic niche conditioning (18, 44). The translational relevance of these programs is further reinforced by the growing therapeutic interest in stromal normalization and *FAP*-centered targeting approaches (62, 63). These findings suggest that nodal dissemination in LSCC may be facilitated by a CAF-conditioned microenvironment that precedes or accompanies tumor cell spread.

This question may be particularly relevant to supraglottic LSCC, where richer lymphatic drainage could amplify the biological consequences of lymphovascular stromal remodeling. Comparative studies across laryngeal subsites are needed to determine whether stromal predictors of nodal spread differ between glottic and supraglottic tumors.

### 6.3 CAFs in radiotherapy and chemoradiotherapy resistance under larynx-preservation settings

In LSCC, the clinical imperative of larynx preservation makes CAF-mediated treatment resistance especially important. Standard organ preservation regimens rely not only on tumor cytotoxicity but also on favorable immune and stromal remodeling after therapy (3, 4). Several distinct mechanisms have been implicated in connecting fibroblast biology to treatment failure.

Ionizing radiation can activate TGF-β and promote NOX4-dependent myofibroblast differentiation, thereby reinforcing fibrosis and tissue stiffening (34, 55). CAF-derived paracrine signals, including *EGFR* ligands, can protect tumor cells from radiation-induced apoptosis in HNSCC models (14). Meanwhile, tumor cells in partial epithelial-to-mesenchymal transition states may exploit matrix-mediated signaling to enhance DNA repair, stress adaptation, and survival (45). These data support the concept that CAF-rich LSCC lesions may be biologically predisposed to incomplete chemoradiotherapy response, even when organ preservation protocols are successfully delivered.

### 6.4 CAFs and resistance to immunotherapy

Immunotherapy resistance in fibroblast-rich tumors is likely multi-layered. Independent translational studies and early clinical data support this concept (13, 39, 62). MyCAF-rich, immune-excluded architecture may limit checkpoint responsiveness. In recurrent or metastatic HNSCC, pembrolizumab-based treatment produces meaningful but still limited response rates overall, and not all PD-L1-positive tumors derive durable benefit (64, 65).

The likely resistance mechanisms are multilayered. They include physical exclusion of T cells by dense extracellular matrix and functional impairment mediated by stromal checkpoint ligands such as Gal9 and possibly PD-L1. Additional contributors are myeloid skewing driven by IL-6, CXCL12, and related inflammatory circuits, oxidative and metabolic suppression of effector immunity, and post-treatment stromal reprogramming toward a more fibrotic residual disease state.

In LSCC, these mechanisms are highly plausible but only partially validated directly. CAF-rich stroma most likely constitutes a biologically distinct immunotherapy resistance program. This conclusion warrants dedicated validation and biomarker-guided combination strategies in laryngeal cancer.

## 7 Therapeutic vulnerabilities and biomarker-guided stromal interventions

CAF targeting approaches can be organized into three mechanistic aims (27–29). These are CAF depletion, CAF normalization, and CAF function blockade. This framework is particularly useful in LSCC, where indiscriminate stromal depletion may be undesirable given the functional complexity of laryngeal tissues and the cautionary lessons from other tumor types (31, 32).

### 7.1 *FAP*-directed strategies

*FAP* remains one of the most clinically advanced CAF-associated targets across solid tumors (63). *FAP*-directed strategies include CAR T cells, bispecific antibodies, antibody-drug conjugates, and *FAP*-targeted imaging approaches that may aid patient stratification. In LSCC, *FAP* is attractive because it marks matrix-active fibroblast programs associated with metastasis-related stromal remodeling (6). However, *FAP* is not expressed across all CAF states. Depletion strategies also raise concerns about effects on wound healing and normal reparative stroma. Accordingly, *FAP*-directed approaches in LSCC may be better suited to selective or biomarker-enriched trials. Nonselective anti-stromal therapy appears less appropriate.

### 7.2 NOX4 inhibition as a CAF-normalizing strategy

NOX4 is among the best-validated CAF-normalizing targets in the context of HNSCC. It is required for TGF-β-driven myofibroblastic differentiation and helps maintain αSMA expression, collagen deposition, and fibrotic immune exclusion (34, 55). Pharmacologic NOX4 inhibition may do more than prevent CAF activation. Preclinical studies suggest partial reversion of established myofibroblastic programs. CD8-positive T-cell infiltration may also improve.

The selective *NOX1/4* inhibitor setanaxib has generated substantial translational interest. An early randomized phase II conference report examined recurrent or metastatic HNSCC with elevated CAF burden. It suggested improved progression-free and overall survival for setanaxib plus pembrolizumab relative to pembrolizumab alone, together with increased intratumoral CD8-related readouts (62). These data are noteworthy because they provide early clinical support for a CAF-normalization strategy. At the same time, broader peer-reviewed confirmation remains important before overextending conclusions. NOX4 inhibition remains a strong candidate stromal strategy for biomarker-enriched LSCC trials. It may be especially relevant in fibrotic, immune-excluded disease.

### 7.3 Blocking stromal signaling hubs: TGF-β, *CXCL12*-*CXCR4*, and IL-6-*JAK*-*STAT3*

Among stromal programs that shape immune dysfunction in LSCC, three signaling axes appear especially relevant. These are TGF-β, *CXCL12*-*CXCR4*, and *IL-6-JAK-STAT3*. TGF-β occupies a central position within CAF-driven stromal remodeling by promoting myofibroblastic stabilization, extracellular matrix accumulation, regulatory T-cell induction, and immune exclusion (46, 47, 52). Together, these effects reinforce both the structural and immunological barriers that restrict effective antitumor immunity. In the context of LSCC, this pathway is of particular interest because it may operate at the interface between constitutive stromal activation and treatment-related fibrotic remodeling. This positioning suggests a dual role in baseline microenvironmental organization and therapy-induced stromal adaptation. The *CXCL12-CXCR4* axis represents a second major stromal signaling node with direct relevance to immune spatial control. It contributes to the sequestration of T cells within stromal compartments while simultaneously supporting the recruitment and retention of suppressive myeloid populations (42, 43). From a translational perspective, this raises the possibility that *CXCR4* antagonism could be used to relieve stromal immune trapping and improve the access of effector lymphocytes to tumor nests, particularly in combination with immune checkpoint blockade. A third convergent pathway, *IL-6*-*JAK*-*STAT3*, links inflammatory fibroblast states to myeloid-biased immunosuppression and cytokine amplification. This sustains a feed-forward inflammatory circuit that may be especially relevant in iCAF-enriched, myeloid-dominant LSCC ecosystems (49, 50). More broadly, experience from head and neck radiotherapy-based combination strategies, including cetuximab-associated radio-enhancement, has underscored an important principle. Signaling interactions involving non-malignant compartments can substantially influence therapeutic response, even when the intervention itself is not CAF-specific (66). These three axes can therefore be viewed not as isolated pathways but as integrated stromal signaling hubs that coordinate matrix remodeling, immune exclusion, and suppressive cell-state reinforcement. As such, they represent rational candidates for stromal-immune combination strategies in LSCC.

### 7.4 Stromal checkpoint-like targets: PD-L1 and Gal9

Because stromal PD-L1 has been observed in LSCC (8, 9), currently approved PD-1/PD-L1-directed agents likely act on both epithelial and stromal checkpoint compartments. However, the specific contribution of CAF-associated PD-L1 to clinical benefit in LSCC is unresolved. Gal9 is even less clinically mature but mechanistically compelling in HNSCC (13). Dual blockade of PD-1/PD-L1 and Gal9-TIM3-related stromal suppression may represent a rational strategy in checkpoint-active, immune-excluded tumors once direct LSCC validation is available.

### 7.5 Extracellular matrix and mechanical niche targeting

The extracellular matrix itself is a therapeutic target. *LOX*/*LOXL2* inhibition, integrin-directed therapies, and discoidin domain receptor modulation may reduce collagen crosslinking, matrix stiffness, and mechanosignaling-driven tumor-stromal reciprocity (40, 41). In LSCC, these approaches may be especially relevant at the invasive front and the putative cartilage-interface niche, where mechanical barriers and tissue architecture are central to disease progression.

### 7.6 Targeting CAF-derived extracellular vesicles and stromal RNA circuits

The LSCC-specific exosomal microRNA data provide a rationale for targeting extracellular vesicle biogenesis or cargo delivery (10, 60). Conceptual approaches include inhibition of neutral sphingomyelinase or Rab-related extracellular vesicle pathways, restoration of tumor-suppressive microRNAs, and antisense oligonucleotide targeting of oncogenic non-coding RNA circuits (67). Nonetheless, these strategies remain early-stage in LSCC. At present, the extracellular vesicle/RNA domain is best regarded as a promising translational frontier rather than a near-clinical platform, with OSCC-derived EV-modulation concepts providing indirect but useful mechanistic context (61).

### 7.7 Rational combination design in LSCC

**Figure 3:**
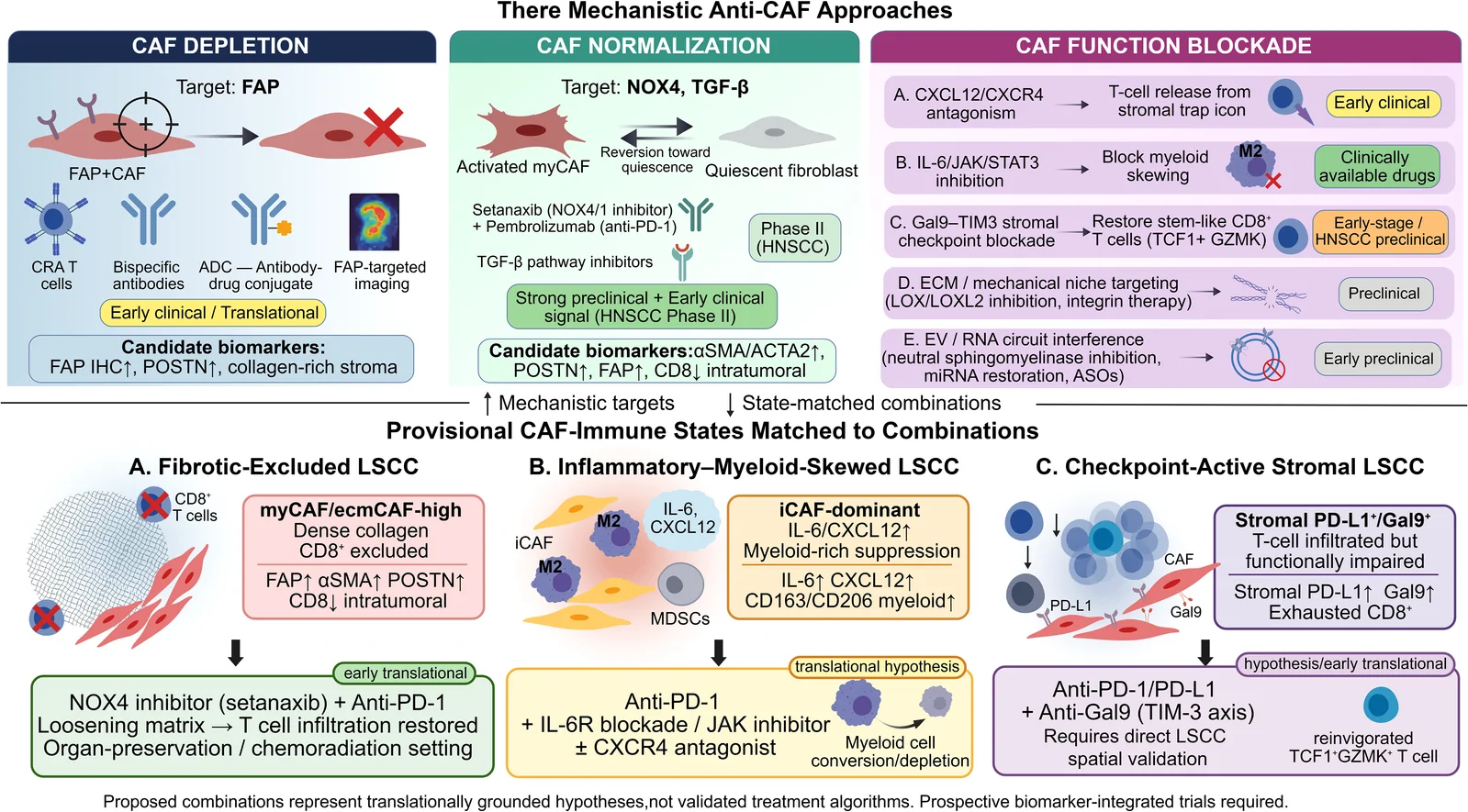
Therapeutic vulnerabilities and biomarker-guided stromal strategies in LSCC. Upper panel: major anti-CAF strategies, including CAF depletion, CAF normalization, and CAF function blockade. Lower panel: proposed biomarker-guided combination strategies matched to provisional CAF-immune phenotypes in LSCC. Representative targets and relative development maturity are indicated. Created with BioGDP.com (38).

In LSCC, combination strategies should be guided by stromal state. A uniform CAF targeting approach is less plausible because fibroblast programs are heterogeneous and immunologically distinct. Rather than treating CAFs as a single therapeutic entity, a more rational framework is to align intervention design with dominant stromal phenotypes, immune architecture, and clinical context. Within this perspective, several biomarker-guided therapeutic paradigms warrant priority investigation. In fibrotic, myCAF-enriched, and immune-excluded tumors, particularly in organ-preservation settings where radiotherapy or chemoradiotherapy remains central, NOX4 inhibition may be added to anti-PD-1-based strategies. This combination may offer a means of loosening matrix-dense stromal barriers and improving immune access. By contrast, some tumors are characterized by checkpoint-active stromal niches, in which T-cell infiltration is present but functionally constrained rather than completely absent. In such tumors, combining anti-PD-1/PD-L1 therapy with stromal checkpoint blockade, such as anti-Gal9-directed approaches, may be more appropriate for reversing local immune paralysis. In iCAF-dominant and myeloid-skewed lesions, inflammatory cytokine circuits contribute to suppressive immune conditioning. In this context, combinations pairing anti-PD-1 with IL-6 receptor blockade or JAK inhibition may be particularly relevant for disrupting fibroblast-driven inflammatory amplification and myeloid-mediated resistance. A further scenario involves stemness-associated, highly plastic, or minimal residual disease states, in which fibroblast-derived extracellular vesicle signaling and RNA-mediated intercellular communication may play a disproportionate role. In such settings, systemic therapy combined with extracellular vesicle interference or RNA-pathway-targeted strategies may represent a promising direction. These proposed combinations remain biologically testable hypotheses, not validated treatment algorithms. Their clinical relevance in LSCC will depend on prospective biomarker integration, spatial patient stratification, and careful evaluation in mechanism-oriented studies. These biomarker-guided stromal vulnerabilities and rational CAF-immune combination strategies are summarized in **Figure 3** and **Table 3**.

**Table 3:** Therapeutic vulnerabilities and biomarker-guided stromal strategies in LSCC.

Strategy	Mechanistic aim	Most relevant CAF-immune context	Development maturity	LSCC evidence status	Candidate biomarkers for selection
*FAP*-directed strategies	Selective depletion/targeting of activated fibroblasts	ecmCAF-high, *FAP*-rich fibrotic tumors	Early clinical/translational across cancers	Indirect to moderate	FAP, POSTN, COMP, collagen-rich stroma, C1QC/SPP1 macrophage proximity
NOX4 inhibition	CAF normalization; reversal of myofibroblastic activation	myCAF-high, fibrotic-excluded tumors	Strong preclinical; early clinical HNSCC signal	High translational relevance, not LSCC-specific clinically	αSMA, *POSTN*, *FAP*, low intratumoral CD8
TGF-β blockade	Reduce matrix barrier, regulatory T-cell induction, myCAF maintenance	myCAF-dominant immune exclusion	Preclinical to early translational	Indirect but strong mechanistic support	TGF-β signature, collagen density, invasive-front fibrosis
*CXCL12/CXCR4* antagonism	Release T-cell trapping; reduce myeloid-derived suppressor cell recruitment	iCAF-rich or stromally trapped T-cell lesions	Early clinical/translational	Indirect to moderate	*CXCL12, CXCR4*, stromal T-cell marginalization
*IL-6/JAK/STAT3* inhibition	Block inflammatory myeloid skewing	iCAF-dominant, myeloid-rich tumors	Clinically available drugs; limited LSCC-specific biomarker data	Indirect	*IL-6, pSTAT3*, CD163/CD206 myeloid enrichment
Stromal checkpoint targeting	Reverse stromal checkpoint-like suppression	Checkpoint-active stromal tumors	PD-1/PD-L1 clinically established; Gal9 early-stage	PD-L1 partially direct; Gal9 indirect in LSCC	Stromal PD-L1, Gal9, exhausted CD8 phenotype
Extracellular matrix/mechanical niche targeting	Reduce stiffness and mechanosignaling	ecmCAF/myCAF-rich invasive-front disease	Preclinical/translational	Indirect	collagen density/alignment, LOX/LOXL2, POSTN/COMP, ITGA6-related tumor-matrix signaling, cartilage-adjacent fibrosis
Extracellular vesicle/RNA-targeted strategies	Interrupt fibroblast-derived extracellular vesicle cargo and non-coding RNA signaling	Stemness-associated, residual, or plastic tumors	Early preclinical	Direct LSCC biological basis but not clinically mature	CAF-derived extracellular vesicle microRNA profile, exploratory RNA markers

## 8 Translational biomarkers and clinical perspectives

### 8.1 Tissue biomarkers for CAF-rich and immune-excluded LSCC

A practical LSCC stromal biomarker panel should integrate CAF-state markers with immune-contexture readouts. Useful components include *FAP*, αSMA, PDGFRβ, *POSTN*, *COL1A1*, CD8, PD-L1, and Gal9. These markers should not, however, be interpreted in isolation. For example, high *FAP*/*POSTN* with low intratumoral CD8 density suggests a fibrotic-excluded phenotype, whereas elevated inflammatory cytokine signatures with abundant myeloid infiltrates may indicate an iCAF-dominant suppressive niche.

Because stromal PD-L1 and Gal9 have uncertain cellular sources, multiplex methods are preferable when feasible. These include multiplex immunohistochemistry, multiplex immunofluorescence, and spatial transcriptomics. More broadly, the predictive value of CAF markers is likely to depend not only on abundance but also on topography, particularly at the invasive front, perivascular compartment, and tumor-cartilage interface. Conceptually, these readouts should be interpreted within broader immune-contexture and tumor immune phenotype frameworks that emphasize not only immune-cell abundance but also spatial organization and functional state (68–74).

### 8.2 Single-cell and spatial signatures for patient stratification

Current evidence suggests that LSCC may be provisionally organized into several recurrent CAF-immune states. These states could provide a useful framework for biologically informed patient stratification, particularly as single-cell and spatial profiling technologies become increasingly integrated into translational research. One such state is a fibrotic-excluded phenotype, characterized by a high burden of myCAF and ecmCAF programs, dense collagen-rich stroma, and limited effective T-cell penetration into tumor nests. This pattern is consistent with a microenvironment in which matrix architecture and fibroblast-mediated contractile remodeling function as major barriers to antitumor immunity. In this setting, therapeutic benefit may be more likely to emerge from stromal normalization or barrier-disrupting approaches designed to improve immune access. A second provisional state is an inflammatory-myeloid-skewed phenotype, marked by enrichment of iCAF programs, elevated IL-6- and CXCL12-associated signaling, and abundant suppressive myeloid infiltrates. This state suggests a microenvironment dominated by cytokine amplification and fibroblast-myeloid crosstalk rather than purely physical exclusion. Such tumors may be especially suitable for combination strategies targeting inflammatory cytokine networks or myeloid-dependent suppressive circuits. A third state may be defined as checkpoint-active stromal LSCC, in which T-cell infiltration is not entirely absent but remains functionally constrained in association with stromal PD-L1 expression and potentially Gal9-related immunoregulatory programs. This pattern indicates a form of spatially retained yet ineffective antitumor immunity. This phenotype may be particularly relevant to dual-checkpoint strategies or therapeutic approaches aimed at stromal normalization in combination with immune reinvigoration. Although these categories remain provisional, they offer a conceptually useful structure for research-level stratification and hypothesis generation. At present, these categories should not be regarded as clinically deployable classification systems. Formal prospective validation, reproducible biomarker definition, and cross-platform standardization are still required. Only then can these CAF-immune states be incorporated into patient selection or treatment decisions.

### 8.3 Liquid biopsy opportunities

CAF-derived exosomal microRNAs are attractive liquid-biopsy candidates in LSCC, particularly given the disease-specific observation of altered exosomal microRNA profiles in supraglottic LSCC-associated fibroblasts (10). Circulating exosomal microRNAs could potentially serve as noninvasive indicators of stromal activation, treatment response, or early relapse, consistent with broader exosome biology across cancers (60, 75). However, it remains unclear whether currently identified LSCC-associated microRNA changes are sufficiently robust, specific, and reproducible for clinical use. Prospective longitudinal studies are needed.

### 8.4 Toward a multidimensional stromal-immune integration framework

To support integrated interpretation of stromal-immune organization in LSCC, we propose a conceptual, multidimensional framework for stromal-immune integration. This framework is intended purely as a hypothesis-generating lens for future translational research, rather than a scoring instrument. Rather than relying on any single marker, this framework aims to capture several biologically interconnected dimensions that are likely to influence the functional impact of CAFs on tumor immunity and therapeutic responsiveness. A first component is stromal burden, reflecting the quantitative extent of *FAP*-rich and αSMA-rich stromal compartments as a surrogate of overall fibroblast-associated microenvironmental dominance. A second component is CAF-state composition, which considers the relative enrichment of distinct fibroblast programs. This includes the balance between myCAF-associated contractile and fibrotic states, iCAF-associated inflammatory states, and extracellular matrix-remodeling programs, all of which may differentially shape immune accessibility and suppression. A third dimension is immune exclusion, defined by the spatial relationship between CAF-enriched regions and CD8-positive T-cell localization, with particular emphasis on whether effector lymphocytes are able to penetrate tumor nests or remain confined to stromal margins. A fourth dimension is therapy context, including prior exposure to radiotherapy, chemoradiotherapy, or PD-1 pathway blockade, since treatment history may substantially reshape fibroblast states, stromal architecture, and immune geography. This integration framework is strictly a conceptual research lens rather than a clinically deployable tool or scoring system. At its present stage, its principal value lies in encouraging a multidimensional view of LSCC biology that integrates stromal abundance, fibroblast state, immune spatial distribution, and prior therapeutic pressure. This approach is preferable to inferring biological behavior or treatment sensitivity from any single biomarker in isolation. Before this conceptual framework could inform any quantitative or clinically deployable tool, it would require translation into an operationalized model, potentially including a formal scoring system. This would involve retrospective derivation in well-annotated cohorts, external validation across independent datasets and analytical platforms, and prospective testing to determine whether it can reproducibly stratify patients or inform treatment selection.

## 9 Discussion

### 9.1 What is established in LSCC, and what is extrapolated?

A balanced appraisal of the literature reveals a clear gradient of evidential certainty across the claims advanced in this field.

Several findings are relatively well supported within LSCC itself. These include the clinical relevance of the laryngeal stromal microenvironment, direct single-cell evidence for fibroblast-associated remodeling in metastatic LSCC, and stemness-related transcriptomic programs that interact with stromal contexts. They further include stromal PD-L1 associations with immune contexture and clinicopathologic variables, and altered CAF-derived exosomal microRNA profiles in supraglottic LSCC (5–10).

A second tier of evidence is strongly supported by data from HNSCC and HPSCC yet has not been fully established in LSCC. This tier comprises robust myCAF/iCAF/ecmCAF state architecture, TGF-β- and CXCL12-driven immune exclusion, Gal9-associated immune-trapping fibroblast programs, IL-6/JAK/STAT3- and GDF15-related myeloid remodeling, and NOX4 inhibition as a CAF-normalizing strategy (11–19, 34, 42, 43, 46, 47, 49, 50, 55, 62).

A third category of claims remains hypothesis-generating in LSCC, including direct confirmation of an MHC-I-high Gal9^+^ CAF state in laryngeal tumors, a dedicated cartilage-interface fibrotic niche resolved at high spatial resolution, and clinically deployable CAF-immune scoring systems.

Recognizing this evidential hierarchy strengthens the translational value of the field by preventing overinterpretation and clarifying which hypotheses are sufficiently mature for prospective experimental and clinical investigation.

### 9.2 Major controversies

Several controversies remain central. First, with respect to taxonomy versus plasticity, whether LSCC requires a site-specific CAF classification distinct from broader HNSCC frameworks remains unresolved. Second, apCAF function remains controversial, as antigen-presenting fibroblasts may be tolerogenic in one context and immunologically permissive in another. Third, depletion versus normalization remains a strategic question. Experience from other tumor types suggests that indiscriminate stromal ablation can be harmful, supporting a preference for normalization or selective functional blockade. Fourth, spatial uncertainty remains a major limitation in LSCC, where key niches, especially cartilage-adjacent and post-treatment fibrotic regions, have not yet been comprehensively mapped. Fifth, subsite heterogeneity remains understudied. Glottic, supraglottic, and transglottic tumors likely differ in baseline lymphatic architecture, stromal composition, and metastatic potential. Studies in lung cancer further show that fibroblast subpopulations can carry site-specific and context-specific prognostic information (76, 77). These findings caution against a universal CAF taxonomy.

### 9.3 Methodological priorities

The field would benefit most from several complementary methodological advances. With respect to tissue acquisition and processing, paired single-cell profiling of treatment-naïve and post-treatment specimens, including salvage laryngectomy tissue, is needed to resolve therapy-induced stromal dynamics. In parallel, cartilage-adapted dissociation and sampling protocols would substantially reduce the chronic underrepresentation of peritumoral stromal compartments. At the level of study design, explicit subsite-aware stratification distinguishing glottic, supraglottic, and transglottic tumors is essential to prevent confounding from anatomically heterogeneous cohort pooling. In parallel, outcome-linked organ-preservation cohorts should be adequately powered to determine whether CAF-rich or immune-excluded states independently predict larynx-preservation failure. High-resolution spatial validation using platforms such as Visium HD, CODEX, MERFISH, or related multiplex approaches will be required to map stromal-immune interactions at subcellular resolution within intact tissue architecture. Direct functional characterization through patient-derived CAF-T-cell, CAF-macrophage, and CAF-tumor co-culture systems will further be indispensable for establishing mechanistic causality beyond correlative association. Collectively, all of these advances will depend on coordinated international biobanking initiatives with rigorous clinicopathologic annotation to ensure the statistical power and biological generalizability necessary for meaningful clinical translation.

From a clinical perspective, CAF heterogeneity should be identified through a staged biomarker workflow rather than by a single CAF marker. A feasible first layer is routine pathology plus multiplex IHC or immunofluorescence on FFPE tissue, combining FAP, αSMA, POSTN/COL1A1, COMP, and PDGFRβ with immune markers including CD8, FOXP3, CD68/CD163/CD206, C1QC or SPP1, PD-L1, and Gal9. A second layer should quantify spatial metrics, including CAF-rich invasive-front stroma, collagen density or alignment, intratumoral versus stromal CD8 localization, and CAF-macrophage proximity. A third, trial-level layer may incorporate targeted RNA panels, spatial transcriptomics, or FAP-directed imaging to assign tumors to operational phenotypes as follows. MyCAF/ecmCAF-high immune-excluded tumors may be prioritized for stromal normalization or ECM-targeted strategies, iCAF/myeloid-rich tumors for IL-6/JAK/STAT3 or CXCL12/CXCR4 blockade, checkpoint-active stromal tumors for PD-1/PD-L1 plus stromal checkpoint approaches, and FAP-high lesions for FAP-directed trials. This workflow remains investigational and requires prospective validation before guiding LSCC treatment.

## 10 Conclusions

CAFs in LSCC should no longer be viewed as a homogeneous stromal backdrop. Instead, they represent dynamic programs that shape matrix architecture, immune geography, treatment response, and possibly larynx preservation outcomes. The current evidence base is already sufficient to support biomarker-driven, stromal-informed translational research. At the same time, the field has not yet matured to the point where all CAF states or stromal checkpoint mechanisms can be considered definitively established in LSCC. The most productive path forward involves evidence-stratified validation, with the most relevant cross-site hypotheses tested directly in LSCC tissues. Spatial and functional approaches should be integrated with rational combination studies matched to dominant CAF-immune states. If successful, this strategy could convert stromal complexity from a barrier into a therapeutic opportunity in laryngeal cancer.

## Glossary

ACTA2: actin alpha 2, smooth muscle
AKT: protein kinase B
apCAF: antigen-presenting cancer-associated fibroblast
CAF: cancer-associated fibroblast
CAR: chimeric antigen receptor
CCL2: C-C motif chemokine ligand 2
CCL5: C-C motif chemokine ligand 5
CCND1: cyclin D1
CD44: cluster of differentiation 44
CD74: cluster of differentiation 74
CDK6: cyclin dependent kinase 6
COL11A1: collagen type XI alpha 1 chain
COL1A1: collagen type I alpha 1 chain
CXCL1: C-X-C motif chemokine ligand 1
CXCL10: C-X-C motif chemokine ligand 10
CXCL12: C-X-C motif chemokine ligand 12
CXCL5: C-X-C motif chemokine ligand 5
CXCL9: C-X-C motif chemokine ligand 9
CXCR4: C-X-C motif chemokine receptor 4
ecmCAF: extracellular matrix-remodeling cancer-associated fibroblast
EGFR: epidermal growth factor receptor
FAP: fibroblast activation protein alpha
FOXP3: forkhead box P3
FSP1: fibroblast-specific protein 1
Gal9: galectin-9
GDF15: growth differentiation factor 15
GZMK: granzyme K
HIF-1α: hypoxia-inducible factor 1-alpha
HNSCC: head and neck squamous cell carcinoma
HPSCC: hypopharyngeal squamous cell carcinoma
iCAF: inflammatory cancer-associated fibroblast
IHC: immunohistochemistry
IL-10: interleukin 10
IL-6: interleukin 6
IL-8: interleukin 8
JAG1: jagged 1
JAK: Janus kinase
lncRNA: long non-coding RNA
LOX: lysyl oxidase
LOXL2: lysyl oxidase like 2
LSCC: laryngeal squamous cell carcinoma
MHC: major histocompatibility complex
MMP11: matrix metallopeptidase 11
myCAF: myofibroblastic cancer-associated fibroblast
NKG2D: natural killer group 2D
NOTCH4: notch receptor 4
NOX1/4: NADPH oxidase 1/4
Nrf2: nuclear factor erythroid 2-related factor 2
PD-1: programmed cell death protein 1
PD-L1: programmed death-ligand 1
PDGFRβ: platelet-derived growth factor receptor beta
PI3K: phosphoinositide 3-kinase
POSTN: periostin
PTEN: phosphatase and tensin homolog
S100A4: S100 calcium-binding protein A4
scRNA-seq: single-cell RNA sequencing
SOX4: SRY-box transcription factor 4
STAT3: signal transducer and activator of transcription 3
TAGLN: transgelin
TCF1: T cell factor 1 (encoded by TCF7)
TGF-β: transforming growth factor beta
TIM3: T-cell immunoglobulin and mucin-domain containing-3
TME: tumor microenvironment
VEGF-C: vascular endothelial growth factor C
VEGF-D: vascular endothelial growth factor D
WNT2: Wnt family member 2
αSMA: alpha-smooth muscle actin
